# Thyroid Isthmus Length and Iodine Turnover as Predictors of Successful Radioactive Iodine Therapy in Patients with Graves' Disease

**DOI:** 10.1155/2017/7354673

**Published:** 2017-12-05

**Authors:** Se Hee Park, Sena Hwang, Seunghee Han, Dong Yeob Shin, Eun Jig Lee

**Affiliations:** ^1^Division of Endocrinology and Metabolism, Department of Internal Medicine, Yonsei University College of Medicine, Seoul 03722, Republic of Korea; ^2^Graduate School, Yonsei University College of Medicine, Seoul 03722, Republic of Korea; ^3^Chaum Life Center, CHA University School of Medicine, Seoul 06062, Republic of Korea; ^4^Division of Endocrinology, Department of Internal Medicine, Inseong Hallym Hospital, Incheon 21079, Republic of Korea

## Abstract

Radioactive iodine (RAI) therapy is an effective treatment option for Graves' disease. However, predicting treatment failures after RAI therapy remains controversial. The objective of this study was to investigate the factors associated with the success rate of RAI therapy for treatment of Graves' hyperthyroidism. Thyroid functional outcome, pre-RAI ultrasonographic features, and clinical parameters were evaluated retrospectively in 98 patients followed up for at least 12 months after RAI (mean RAI dose was 11.7 ± 1.8 mCi). Hypothyroidism was achieved in 59 patients (60.2%), and euthyroidism in 16 patients (16.3%), while 23 patients (23.5%) remained hyperthyroid. Age, sex, body mass index, pre-RAI thyroid function, or thyroid-stimulating immunoglobulin levels were not associated with treatment outcome. Length of thyroid isthmus (*p* = 0.028) and 2- to 24-hour iodine uptake ratios (*p* = 0.002) were significantly associated with treatment failure, which was defined as a persistent hyperthyroid status after RAI therapy. Patients with a longer isthmus had a higher risk of remaining hyperthyroid, with a threshold for isthmus length of 5.2 mm, with a sensitivity of 69.6% and specificity of 70.3% for treatment success. Measuring the length of the thyroid isthmus can be a simple and useful way to predict RAI treatment outcome.

## 1. Introduction

Graves' disease is an autoimmune thyroid disease caused by the binding of stimulating antibodies against TSH receptors (TRAbs) to TSH receptors on thyroid cells. This binding stimulates follicular hypertrophy and hyperplasia, causing thyroid enlargement as well as increased thyroid hormone production [1]. Graves' disease accounts for 60%–80% of patients with hyperthyroidism, depending on regional factors, especially iodine intake [2]. It occurs more commonly in women than in men [3]. Untreated hyperthyroidism can lead to osteoporosis and cardiovascular complications such as atrial fibrillation and heart failure, and severe hyperthyroidism or thyroid storm is associated with a mortality rate of 20–50% [4]. To date, the three main treatment options for Graves' disease are antithyroid drugs, thyroidectomy, and radioactive iodine (RAI) therapy. Although the efficacies are similar among all three treatments, usually, antithyroid drugs are associated with the higher recurrence rate of the disease, compared to other therapeutic modalities. [5].

RAI therapy has been used since the 1940s and is the most preferred treatment in the United States [6]. It is also effective for recurrent Graves' disease, but the appropriate dose of ^131^I is often based on empirical content, and predictive factors of therapeutic effects have not yet been fully elucidated [7]. Some studies attempting to calculate the appropriate ^131^I dose based on thyroid size and 24-hour iodine uptake have reported increased efficacy over fixed dose [8]. However, other studies have shown that administration of a fixed dose of ^131^I is effective, as it simplifies procedures, reduces costs, and makes procedures more efficient [9, 10]. It is also important to predict the success of treatment and improvement in thyroid function after RAI therapy. Even though some factors such as age, sex, size of thyroid gland, degree of hyperthyroidism before treatment, and rate of iodine uptake by the thyroid gland have been suggested as predictors of successful RAI treatment, it continues to remain controversial [11, 12].

Thyroid gland volume, which is known to be the most important factor in determining the dose and predicting the success of treatment, is calculated mainly using ultrasonography, and this method is known to be relatively accurate [13]. However, as Graves' disease is characterized by thyroid enlargement, thyroid gland volume can be cumbersome to measure with a conventional probe and may be less accurate. Because the length of the thyroid isthmus correlates with the volume of the thyroid gland [14] and is easy to measure, it can be used as an indicator before RAI treatment. The purpose of this study was to analyze the associated indicators and usefulness of thyroid isthmus length as a predictor of RAI therapy in Graves' disease.

## 2. Materials and Methods

### 2.1. Study Populations

Patients treated with RAI for Graves' disease at Severance Hospital from January 2010 to December 2013 were followed up for more than 12 months, and we were able to retrospectively evaluate the pretreatment thyroid volume in 98 patients by using cervical ultrasonography.

Patients were treated with a dose of RAI determined semiquantitatively based on thyroid gland volume and the 24-hour RAI uptake rate on ^131^I scans. Patients who were receiving antithyroid drugs had to stop medication one week before the RAI therapy.

Treatment success was assessed using thyroid function tests in these patients for at least twelve months after treatment. RAI treatment outcomes were categorized into three groups, hypothyroid, euthyroid, and hyperthyroid, according to thyroid function test results at twelve months after treatment. A hypothyroid or the euthyroid status was defined as RAI therapy success, whereas persistent hyperthyroidism was defined as treatment failure. The association between RAI treatment failure and various clinical parameters including age, sex, height, weight, body mass index (BMI), thyroid gland volume, and isthmus length on ultrasonographic images, thyroid function and thyroid autoantibody levels before treatment, dose of RAI, RAI uptake rate, and serum selenium and 25-hydroxycholecalciferol levels was analyzed. This study was approved by the Yonsei University College of Medicine Institutional Review Board (4-2017-0134).

### 2.2. Thyroid Function and Antithyroid Antibody Test

Serum concentrations of TSH (normal range, 0.35–4.94 IU/mL), free T4 (normal range, 0.70–1.48 ng/dL), and T3 (normal range, 0.58–1.59 ng/mL) for thyroid function evaluation were measured by microparticle chemiluminescence immunoassay (Abbott Ireland Diagnostics Division, Longford, Ireland). TRAb levels were measured by two different methods: M22-TRAb (TRAb) was measured by a third-generation TBII electrochemiluminescence immunoassay (Elecsys/Cobas; Roche Diagnostics, Mannheim, Germany) and Mc4-TSAb (thyroid-stimulating immunoglobulin, TSI) was measured by the Thyretain™ TSI reporter BioAssay (Diagnostic Hybrids Inc., Athens, OH, USA). The antibody test was defined as positive when TRAb levels were higher than 1.75 IU/L and Mc4-TSAb was higher than the standard value of the sample ratio of 140%. Concentrations of thyroglobulin antibody (normal range, 0–130.6 IU/mL) and thyroperoxidase antibody (normal range, 0–13.7 IU/mL) as a thyroid autoantibody were measured by electrochemiluminescence immunoassay (Roche Diagnostics, Mannheim, Germany).

### 2.3. Thyroid Volume Measurement

Gray-scale ultrasonography evaluation of the thyroid gland was performed with a 5 to 12 MHz linear transducer (iU22; Philips Medical Systems, Bothell, WA, USA) or a 6 to 13 MHz linear transducer (EUB-7500; Hitachi Medical, Tokyo, Japan). For imaging of the thyroid gland, the patients were instructed to take a supine position with a cushion under the shoulder and neck hyperextended.

The ultrasonographic procedure for the total volume measurement was performed as previously described by Ueda [15]. In other words, each lobe of the thyroid gland was assumed to be a prolate spheroid. We then measured the height (D1), width (D2), and depth (D3) of each lobe. The volume of each lobe was calculated using a standard geometric formula: volume of a prolate ellipsoid = D1 × D2 × D3 × 0.523. The volume of the whole thyroid gland was estimated as the sum of the volume of each lobe.

### 2.4. Definition of Treatment Effect

Hypothyroid patients had a persistently low free T4 concentration and an elevated TSH concentration within six months after therapy and had been on levothyroxine therapy to normalize TSH concentrations. Euthyroidism was defined as serum T4 and TSH concentrations within the normal range without levothyroxine replacement at six months. Hyperthyroidism was defined as free T4 that remained elevated and suppressed TSH or a continued requirement for antithyroid medication.

### 2.5. Statistical Analysis

The variables of each group were compared based on the results of the first radioisotope treatment. Continuous variables are reported as mean ± standard deviation and analyzed by one-way ANOVA. For comparison and analysis of categorical variables, a χ^2^ test was used. The results and the factors affecting these results were analyzed by multivariate logistic regression analysis. Receiver operating characteristic (ROC) curves were used to determine the threshold of thyroid isthmus length or thyroid volume to predict the success of radioisotope therapy. Statistical significance was defined as a *p* value less than 0.05. Statistical analysis was performed using SPSS version 23 (IBM Corp., Armonk, NY, USA).

## 3. Results

### 3.1. Clinical Characteristics of Patients and Analysis of Clinical Factors according to RAI Treatment Results

There were 98 patients (31 men, 67 women) with Graves' disease with a mean age of 44.1 ± 14.0 years. The mean follow-up duration was 33.1 ± 14.0 months. The mean volume of the thyroid gland measured by ultrasonography was 32.3 ± 21.0 cm^3^, and the mean length of the thyroid isthmus was 5.4 ± 3.2 mm. In the thyroid function test before RAI treatment, T3 was elevated to 1.85 ± 0.97 ng/mL (normal range, 0.58–1.59 ng/mL), free T4 was elevated to 2.05 ± 1.22 ng/mL (normal range, 0.70–1.48 ng/dL), and TSH was suppressed to below the measurement range in approximately 77.6% of the patients. TRAb positivity before RAI treatment was found in approximately 88.8% of the patients, and the mean TRAb level was 13.45 ± 14.21 IU/L (normal range, below 1.75 IU/L). The mean dose of RAI administered was 11.7 ± 1.8 mCi (Table 1).

Based on the results of the first RAI treatment, the patients were divided into a hypothyroid group (59 patients, 60.2%), normal function group (16 patients, 16.3%), and persistent hyperthyroid group (23 patients, 23.5%). There was no difference in mean age, sex ratio, follow-up period, height, weight, or BMI among the three groups. The mean thyroid volume was 53.5 ± 27.1 cm^3^ in the hyperthyroid group, which was significantly larger than that in the euthyroid group (29.2 ± 10.6 cm^3^) (*p* = 0.001) and in the hypothyroid group (24.7 ± 13.7 cm^3^) (*p* < 0.001), and the mean length of the thyroid isthmus was the longest (8.0 ± 4.2 mm) in the hyperthyroid group (*p* < 0.001). The mean TRAb level was the highest (20.25 ± 20.08 IU/L) in the hyperthyroidism group, which was significantly higher than that in the euthyroid group (7.98 ± 7.82 IU/L) (*p* = 0.037). However, TSI values did not differ among the three groups (*p* = 0.793), and there was no significant difference in other thyroid autoantibodies. There was no difference in the administered dose of RAI among the three groups (*p* = 0.191). In the ^131^I scan, the 2-hour intake rate was highest in the hyperthyroid group, followed by the hypothyroid group and then the euthyroid group, and the difference was statistically significant (*p* = 0.030). However, with regard to the 24-hour intake rate, there was no significant difference among the three groups. The 2- to 24-hour ^131^I uptake ratio was significantly higher in the persistent hyperthyroid group (*p* = 0.002) (Table 2).

### 3.2. Correlation between Thyroid Isthmus Length and Success of RAI Treatment

**Figure 1:**
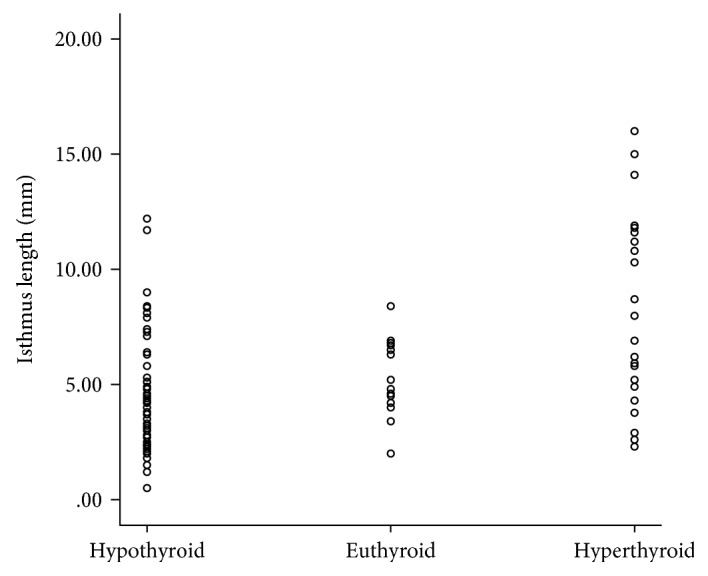
Distribution of isthmus length based on treatment results after radioactive iodine therapy.

**Figure 2:**
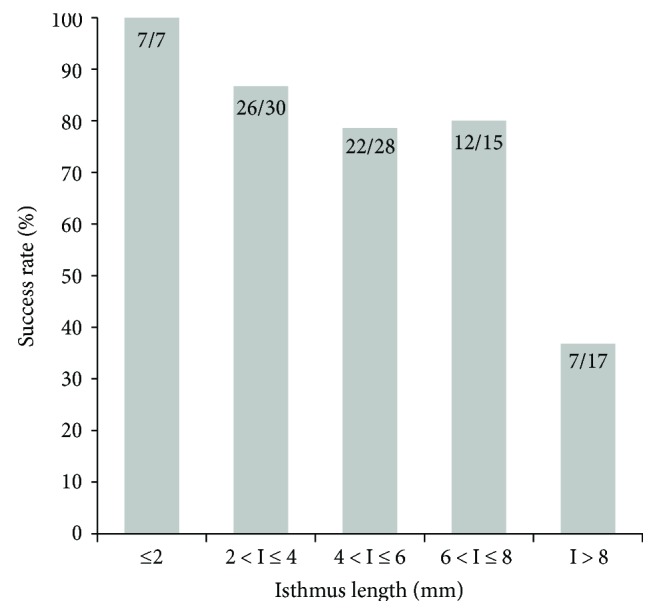
Success rates of radioactive iodine therapy for various isthmus length subgroups in patients with Graves' disease. Data are presented as number of patients with treatment success/total patients.

As shown above, the mean length of the thyroid isthmus was longer in the hyperthyroidism group (8.0 ± 4.2 mm) than in the other groups (*p* < 0.001). The distribution of isthmus length according to treatment results after RAI therapy can be seen in Figure 1. Thus, the longer the isthmus length, the more likely that hyperthyroidism persisted. This tendency was more pronounced when the treatment success rate was compared between groups divided according to isthmus length of 2 mm. When the isthmus length is less than 2 mm, the treatment success rate is close to 100%, but as the isthmus length becomes longer, the success rate gradually decreases and is about 41% when the isthmus length is 8 mm or more (Figure 2). There was also a significant positive correlation between isthmus length and treatment failure (*p* < 0.001, *r* = 0.453).

### 3.3. Threshold Values of Isthmus Length Related to Successful RAI Treatment

**Figure 3:**
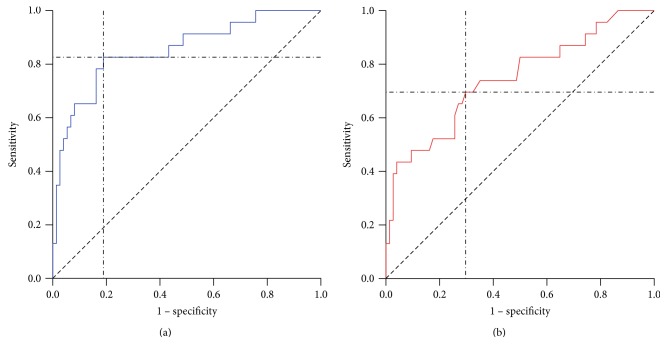
Receiver operating characteristic curve used to determine threshold values of isthmus length related to successful radioactive iodine therapy in patients with Graves' disease. (a) For a volume of 35 cm^3^, sensitivity was 82.6%, and specificity was 81.1% (AUC, 0.852; *p* < 0.001). (b) For an isthmus length of 5.2 mm, sensitivity was 69.6%, and specificity was 70.3% (AUC, 0.746, *p* < 0.001).

To determine the threshold values of isthmus length related to successful RAI in patients with Graves' disease, an ROC curve was used. For a thyroid volume of 35 cm^3^, the sensitivity was 82.6% and the specificity was 81.1% (AUC, 0.852; *p* < 0.001). For an isthmus length of 5.2 mm, the sensitivity was 69.6% and the specificity was 70.3% (AUC, 0.746; *p* < 0.001). We found a failure rate of 41.7% among patients with thyroid isthmus length > 5.2 mm and 13.1% in those with thyroid isthmus length ≤ 5.2 mm (*p* = 0.001). The overall treatment success rate was 76.5% (Figure 3).

### 3.4. Factors Associated with Treatment Failure after RAI Treatment

Multivariate logistic regression analysis was used to identify variables related to RAI treatment failure. An isthmus length > 5.2 mm (OR, 4.50; 95% CI, 1.18–17.24; *p* = 0.028) and a 2- to 24-hour iodine uptake rate more than 0.8 (OR, 8.10; 95% CI, 2.10–31.23; *p* = 0.002) were significantly associated with treatment failure defined as a persistent hyperthyroid status after RAI therapy. There were no significant differences in age, sex, administered ^131^I dose, or TRAb level (Table 3). When thyroid volume was analyzed in the same model, instead of isthmus length, thyroid volume > 35 cm^3^ (OR, 11.89; 95% CI, 2.63–53.68; *p* = 0.001) was significantly associated with persistent hyperthyroid status after RAI therapy.

## 4. Discussion

Our study has shown that an isthmus length of 5.2 mm or more and a 2- to 24-hour iodine uptake rate of 0.8 or more were independent predictors of treatment failure after RAI treatment. The likelihood of a failed first RAI treatment was higher in patients with Graves' disease if the isthmus length was longer. Using an ROC curve, we determined the threshold values of isthmus length related to successful RAI in patients with Graves' disease. On the contrary, the mean level of TRAb was higher in the hyperthyroidism group in univariate analysis, but it was not significant in multivariate analysis.

RAI treatment has been used to treat Graves' disease for the past 60 years and, during that time, has been proven to be both safe and effective, as a primary treatment or when antithyroid drugs are not able to resolve thyrotoxicosis. In the United States, RAI treatment is the most preferred, while in Japan, Korea, and Europe, antithyroid drugs are the preferred treatment [6]. Most patients become euthyroid state, and clinical signs improve within 4–8 weeks after RAI treatment. Hypothyroidism may appear from the fourth week onward, but most often occurs within 2–6 months. The persistence of hyperthyroidism at six months after RAI treatment heralds the probable requirement of an additional dose of RAI treatment. Therefore, identifying patients with a high possibility of RAI treatment failure plays an important role in precise follow-up and additional treatment decisions.

Thyroid ultrasonography is a useful tool for detecting Graves' disease [16]. It is well known that the larger the thyroid volume, the higher is the probability for RAI treatment failure [17, 18]. However, to obtain the thyroid volume, the height, the width, and the depth of thyroid must be measured, and complex formulas need to be used to calculate the thyroid volume. Furthermore, it may be difficult to obtain the thyroid volume in patients whose thyroid is enlarged by goiter. On the contrary, the thyroid isthmus length is easy to measure and correlates with thyroid volume [14]. Therefore, if the relationship between isthmus length and RAI treatment outcome was confirmed, thyroid isthmus length could be a useful clinical parameter in the decision process of RAI treatment. In our study, using the ROC curve, we found that the cutoff value of the isthmus length that increases the probability of treatment failure was 5.2 mm. Therefore, physicians considering RAI therapy for patients with Graves' disease need to carefully measure the length of the isthmus using ultrasonography to predict the therapeutic outcome.

A higher 2- to 24-hour ^131^I uptake ratio in the thyroid gland could reflect a faster iodine turnover in thyroid cells, thus shortening the residual time of therapeutic ^131^I in the thyroid gland and therefore contributing to treatment failure [19]. The time for performing a thyroid scan to measure the iodine uptake ratio varies slightly across studies [18–20]. We used the 2- to 24-hour ^131^I uptake ratio as an index to predict rapid ^131^I turnover, as this is known to be an important factor in predicting the therapeutic outcome. According to multivariate logistic regression, when the 2- to 24-hour ^131^I uptake rate was more than 0.8, the probability of treatment failure was statistically significantly higher than when it was 0.8 or less.

The inverse relationship of high thyroid volumes and RAI treatment failure was first studied by Goolden and Fraser [21], but to the best of our knowledge, there were no studies on the relationship between RAI treatment and thyroid isthmus length. As it is easy to determine isthmus length, it is expected to be highly useful in clinical practice. Additionally, if used together with the 2- to 24-hour ^131^I uptake ratio, a more accurate selection of candidate patients with Graves' disease for whom RAI treatment is more effective and beneficial can be possible.

This study has some limitations that should be addressed. First, the patients involved in this study were from a center in South Korea, where antithyroid drugs are the most commonly used primary treatment for Graves' disease [22]. Therefore, there may have been a selection bias, and the enrolled subjects in this study might be somewhat refractory to the usual antithyroid drug treatment. Further studies are required to confirm these results and to generalize the clinical usefulness of thyroid isthmus length in treatment-naive patients. Second, as a retrospective study, this study was limited in controlling all the clinical parameters confounding RAI treatment efficacy. More prospective controlled studies carried out in larger patient populations in multiple centers are needed. Third, in the measurement of thyroid volume and isthmus length using ultrasonography, minor errors might occur depending on the operator even though objective criteria were used.

## 5. Conclusion

The results from our study are consistent with the results from previous studies showing that increased thyroid volume and a higher ^131^I uptake ratio increased the probability of RAI treatment failure [17, 18]. In addition, we found that the longer the isthmus length, the higher was the probability of RAI treatment failure, with a cutoff value of 5.2 mm. Physicians considering RAI therapy for patients with Graves' disease can adopt this simple and useful parameter as an additional predictive factor of therapeutic outcome.

## Figures and Tables

**Table 1:** Baseline clinical characteristics of patients with Graves' disease.

Characteristic	Total patients (*n* = 98)
Age, years	44.1 ± 14.0
Sex (female, %)	67 (68.4%)
Follow-up duration, months	33.1 ± 14.0
BMI, kg/m^2^	23.4 ± 3.2
Isthmus, mm	5.4 ± 3.2
Thyroid volume, cm^3^	32.3 ± 21.0
Pre-RAI T3, ng/mL	1.85 ± 0.97
Pre-RAI free T4, ng/mL	2.05 ± 1.22
Undetectable TSH (*n*, %)	76 (77.6%)
Pre-RAI TRAb, IU/L	13.45 ± 14.21
Pre-RAI TSI, %	318.5 ± 208.7
RAI dose, mCi	11.7 ± 1.8
2-hour uptake, %	44.3 ± 22.5
24-hour uptake, %	66.0 ± 21.5
2-hour/24-hour ratio	0.65 ± 0.24
Serum selenium, *μ*g/L	118.2 ± 17.1
Serum 25(OH)D, ng/dL	19.0 ± 9.1

Data are presented as mean ± SD or number (%). BMI: body mass index; RAI: radioactive iodine; TRAb: TSH receptor antibody; TSI: thyroid-stimulating immunoglobulin; 25(OH)D: 25-hydroxycholecalciferol.

**Table 2:** Comparison between patients of different outcome groups after the first RAI treatment.

	Hypothyroid	Euthyroid	Hyperthyroid	*p*
Number of patients	59 (60.2%)	16 (16.3%)	23 (23.5%)	
Age, years	43.5 ± 13.5	50.1 ± 17.0	41.4 ± 12.4	0.149
Sex (male : female)	22 : 37	3 : 13	6 : 17	0.297
Follow-up duration, months	34.3 ± 12.7	34.5 ± 13.8	29.2 ± 17.0	0.314
Height, cm	162.9 ± 7.4	159.1 ± 8.0	163.3 ± 7.9	0.236
Weight, kg	61.6 ± 11.4	60.0 ± 8.7	61.6 ± 13.5	0.827
BMI, kg/m^2^	23.3 ± 3.1	23.7 ± 2.7	23.3 ± 3.9	0.931
Isthmus, mm	4.5 ± 2.5	5.2 ± 1.6	8.0 ± 4.2	<0.001
Thyroid volume, cm^3^	24.7 ± 13.7	29.2 ± 10.6	53.5 ± 27.1	<0.001
Pre-RAI T3, ng/mL	1.98 ± 1.15	1.52 ± 0.39	1.75 ± 0.52	0.241
Pre-RAI free T4, ng/mL	1.95 ± 1.14	1.84 ± 1.59	2.47 ± 1.09	0.165
Pre-RAI TRAb, IU/L	12.24 ± 11.77	7.98 ± 7.82	20.25 ± 20.08	0.030
Pre-RAI TSI, %	305.2 ± 213.8	331.9 ± 184.2	343.6 ± 220.0	0.793
Pre-RAI TPO Ab, IU/mL	267.9 ± 241.8	233.5 ± 264.1	356.8 ± 226.1	0.226
Pre-RAI Tg Ab, IU/mL	786.1 ± 1310.8	337.9 ± 690.2	409.1 ± 840.4	0.247
RAI dose, mCi	11.5 ± 1.5	12.8 ± 2.8	11.3 ± 1.5	0.191
2-hour uptake, %	41.8 ± 21.1	38.2 ± 21.3	54.8 ± 24.3	0.030
24-hour uptake, %	66.1 ± 20.8	61.1 ± 22.7	69.1 ± 22.7	0.524
2-hour/24-hour ratio	0.60 ± 0.21	0.60 ± 0.23	0.80 ± 0.26	0.002
Serum selenium, *μ*g/L	121.0 ± 17.5	108.7 ± 14.9	117.4 ± 15.9	0.101
Serum 25(OH)D, ng/mL	18.0 ± 9.2	22.8 ± 10.5	19.1 ± 7.9	0.471

Data are presented as mean ± SD or number (%). One-way ANOVA and χ^2^ tests were performed. BMI: body mass index; RAI: radioactive iodine; TRAb: TSH receptor antibody; TSI: thyroid-stimulating immunoglobulin; TPO Ab: thyroperoxidase antibody; Tg Ab: thyroglobulin antibody; 25(OH)D: 25-hydroxycholecalciferol.

**Table 3:** Multivariate logistic regression analysis of factors associated with persistent hyperthyroid status after radioactive iodine therapy.

Factor	OR (95% CI)	*p*
Age, years	0.99 (0.95–1.05)	0.841
Sex (female)	1.33 (0.30–5.85)	0.711
BMI, kg/m^2^	0.95 (0.77–1.16)	0.592
Isthmus length, mm		
≤5.2	Reference	
>5.2	4.50 (1.18–17.24)	0.028
2-hour/24-hour ^131^I uptake ratio		
≤0.8	Reference	
>0.8	8.10 (2.10–31.23)	0.002
Administered ^131^I dose, mCi	0.85 (0.58–1.25)	0.406
TSH receptor antibody, IU/L	1.01 (0.96–1.06)	0.755

BMI: body mass index.

## Abbreviations

BMI: Body mass index
RAI: Radioactive iodine
Tg Ab: Thyroglobulin antibody
TPO Ab: Thyroperoxidase antibody
TRAb: Thyroid-stimulating hormone receptor antibody
TSI: Thyroid-stimulating immunoglobulin
25(OH)D: 25-hydroxycholecalciferol.
